# A systems-level gene regulatory network model for *Plasmodium falciparum*

**DOI:** 10.1093/nar/gkaa1245

**Published:** 2021-01-15

**Authors:** Maxwell L Neal, Ling Wei, Eliza Peterson, Mario L Arrieta-Ortiz, Samuel A Danziger, Nitin S Baliga, Alexis Kaushansky, John D Aitchison

**Affiliations:** Center for Global Infectious Disease Research, Seattle Children's Research Institute, Seattle, WA, USA; Center for Global Infectious Disease Research, Seattle Children's Research Institute, Seattle, WA, USA; Institute for Systems Biology, Seattle, WA, USA; Institute for Systems Biology, Seattle, WA, USA; Center for Infectious Disease Research, Seattle, WA, USA; Institute for Systems Biology, Seattle, WA, USA; Center for Global Infectious Disease Research, Seattle Children's Research Institute, Seattle, WA, USA; Department of Pediatrics, University of Washington, Seattle, WA, USA; Department of Global Health, University of Washington, Seattle, WA, USA; Brotman Baty Institute, Seattle, WA, USA; Center for Global Infectious Disease Research, Seattle Children's Research Institute, Seattle, WA, USA; Department of Pediatrics, University of Washington, Seattle, WA, USA; Department of Biochemistry, University of Washington, Seattle, WA, USA

## Abstract

Many of the gene regulatory processes of *Plasmodium falciparum*, the deadliest malaria parasite, remain poorly understood. To develop a comprehensive guide for exploring this organism's gene regulatory network, we generated a systems-level model of *P. falciparum* gene regulation using a well-validated, machine-learning approach for predicting interactions between transcription regulators and their targets. The resulting network accurately predicts expression levels of transcriptionally coherent gene regulatory programs in independent transcriptomic data sets from parasites collected by different research groups in diverse laboratory and field settings. Thus, our results indicate that our gene regulatory model has predictive power and utility as a hypothesis-generating tool for illuminating clinically relevant gene regulatory mechanisms within *P. falciparum*. Using the set of regulatory programs we identified, we also investigated correlates of artemisinin resistance based on gene expression coherence. We report that resistance is associated with incoherent expression across many regulatory programs, including those controlling genes associated with erythrocyte-host engagement. These results suggest that parasite populations with reduced artemisinin sensitivity are more transcriptionally heterogenous. This pattern is consistent with a model where the parasite utilizes bet-hedging strategies to diversify the population, rendering a subpopulation more able to navigate drug treatment.

## INTRODUCTION

Despite decades-long eradication campaigns, malaria remains a global burden with an estimated 405 000 deaths worldwide in 2018 (1), largely as a result of the deadliest malaria parasite, *Plasmodium falciparum*. While malaria-associated deaths have declined over the past decade, likely due to both vector control measures and the roll-out of artemisinin-based combination therapies, the annual death rate has plateaued in recent years. The origin of all malaria-associated mortality and morbidity is the destructive, cyclic asexual development of blood stage parasites that leads to erythrocyte death. Thus, elucidating the parasite's molecular regulatory mechanisms during its blood stage provides opportunities for identifying drug targets that would reduce the global burden of the disease. While extensive gene regulation at the level of translational repression occurs during the parasite's mosquito-to-man and man-to-mosquito transitions (2–4), it appears that transcriptional, not translational regulation, plays a dominant role in protein regulation during the asexual, blood stage cycle (5). Transcriptional profiling has illustrated oscillatory patterns in cohorts of genes (6), suggesting that tightly regulated transcriptional networks initiate and/or respond to parasite life cycle progression within the asexual blood stage. However, outstanding questions that surround transcriptional regulation in *P. falciparum* asexual stages remain. To elucidate the gene regulatory interactions that contribute to key cellular processes in blood stage *P. falciparum*, we aimed to build a predictive genome-scale transcriptional regulatory network (TRN) for the parasite that could be applied to laboratory and field-isolated *P. falciparum* strains alike.

TRNs link transcription factors (TFs) with their targets and are generally constructed using a set of transcriptomes and a pre-existing set of TF-target pairs (7). A TRN, therefore, can provide biological insight by predicting—on a global scale—which proteins regulate which genes, which proteins are critical in functional networks, and how the organism's overall regulatory system is organized. The Apicomplexan Apetala2 (ApiAP2) family of DNA binding proteins is the dominant set of characterized TFs for *Plasmodium* (8), and has been demonstrated to bind specific DNA sequences (9) and regulate multiple steps in parasite life cycle progression (8,10) including erythrocyte invasion (11), blood stage replication (8), sexual differentiation (12), oocyst development (13), sporogony (14) and liver stage development (15). Other TFs have been molecularly analyzed (16) and putative TFs have been identified using computational approaches (17,18). Building a *P. falciparum* TRN allowed us to predict and quantify, on a genome scale, which of these transcriptional regulators influence which target genes. Thus, our TRN represents a large set of hypotheses about which gene products regulate the expression of other genes and to what degree. Together, these hypotheses provide a systems-level roadmap that can help guide empirical studies aimed at unraveling the mechanistic relationships between molecular role players in *P. falciparum*.

**Figure 1.**
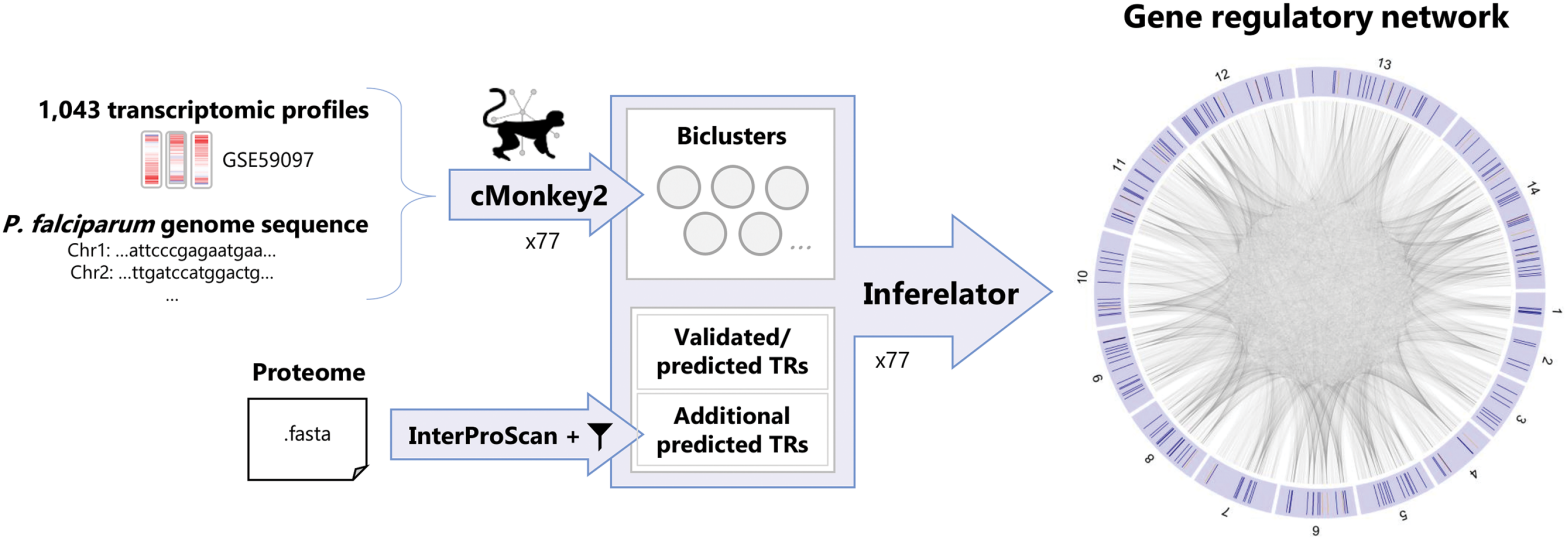
Overall workflow used to generate the global *P. falciparum* EGRIN. We used cMonkey2 to generate biclusters containing genes showing coherence across sample subsets, then used the Inferelator tool to combine that information with a list of transcription regulators (TRs) to generate a genome-scale regulatory network. A higher-resolution image of the network visualization on the right is provided in Supplementary Figure S4. Network visualization generated using the ‘circlize’ R package (69).

To construct the network, we employed a set of systems biology tools and an analysis methodology previously developed for constructing Environmental and Gene Regulatory Influence Networks (EGRINs) in microbes such as *Halobacterium salinarum* (19,20), *Escherichia coli* (19), *Mycobacterium tuberculosis* (21) and *Saccharomyces cerevisiae* (22) (Figure 1). The genome-level transcriptional control networks predicted by these EGRINs have been validated previously through, for example, empirical confirmation of predicted TF binding sites in *E. coli* (19) and gene expression changes due to TF overexpression in *M. tuberculosis* (21). The biological insights derived from these EGRINs include the identification of novel regulators of peroxisome-related genes in *S. cerevisiae* (22) and regulators of bedaquiline tolerance in *M. tuberculosis* (23). We built the *P. falciparum* EGRIN (*Pf*EGRIN) by analyzing transcriptomic data sets to discover co-regulated groups of genes, and then performed regularized regression to determine which proteins regulate those genes, and to what extent. The result is a set of weighted interactions between transcription regulators (TRs) and their targets comprising a genome-wide regulatory network that can be used to predict target gene expression levels based on expression levels of their regulators (Figure 1). Here, we detail the development of the *Pf*EGRIN, report its capabilities for predicting global gene expression across multiple validation data sets, characterize its structural organization, and profile TRs with the highest number of regulatory targets in the model. Moreover, we use the EGRIN model to gain insight into mechanisms that enable artemisinin resistance.

Beyond outputting weighted TR-target interactions (i.e. hypotheses regarding which TRs influence the expression of which transcripts), EGRIN models facilitate the identification of concordant sets of transcripts associated with a given phenotype. A critical phenotype of global importance is the loss of artemisinin sensitivity in *Plasmodium* parasites. Artemisinin and its derivatives are widely used in combination therapies against malaria to quickly reduce the patient's parasite biomass. Several countries have seen the emergence of parasite strains that require extended clearance times following artemisinin-based therapy, threatening one of the cornerstones of malaria treatment across the globe (24). Importantly, although genetic correlates of artemisinin resistance have been identified (25), including mutations in the Kelch13 protein (26,27), not all parasites with a reduction in sensitivity of artemisinin harbor Kelch13 mutations, and the molecular pathways underpinning artemisinin resistance include global processes such as production of phosphatidylinositol 3-phosphate containing vesicles (28), oxidative stress (29), the unfolded protein response (30) and hemoglobin endocytosis (31). A comprehensive view of how a parasite is able to circumvent artemisinin has yet to be fully defined (32). To elucidate properties associated with artemisinin resistance, we identified concordant gene sets generated for the EGRIN that were associated with transcriptomic samples collected prior to artemisinin-based therapy that showed an artemisinin-sensitive (AS) or artemisinin-resistant (AR) phenotype after therapy. We hypothesized that molecular mechanisms underlying artemisinin resistance would manifest as gene sets showing elevated coherence among samples with longer parasite clearance times. Surprisingly, our analysis revealed that artemisinin resistance is linked to dramatically *less* coherence across a range of gene regulatory mechanisms, including—but not limited to—those associated with processes known to be part of the parasite's bet-hedging strategy for evading the immune system during blood stage infection and persisting in the presence of environmental stress (33,34).

## MATERIALS AND METHODS

### Data sources

Transcriptomic data used to train our *Pf*EGRIN model were downloaded from Gene Expression Omnibus (GEO) accession GSE59097 using the R package GEOquery (35). This data set is associated with a study by Mok, *et al.* (30) that examined transcriptomic correlates of artemisinin resistance. It consists of microarray-based gene expression measurements on 1043 *P. falciparum* field isolates obtained in Bangladesh, Democratic Republic of Congo, Cambodia, Laos, Myanmar, Thailand and Vietnam. Each transcriptomic profile in this data set represents an individual patient's infection, was obtained prior to artemisinin-based therapy, and is associated with an infection-specific parasite clearance half-life value. AR and AS parasites were found in all geographic regions where sampling occurred.

The Mok *et al.* study also includes a separate 110-sample transcriptomic data set (GEO accession GSE59098) consisting of microarray measurements on blood stage parasites that were collected from 19 of the Pailin, Western Cambodia patients in accession GSE59097, cultured *ex vivo*, and then sampled at various timepoints over 40 hours. This data set was one of three used to assess the model's ability to predict gene expression levels in transcriptomic data not used to train the model. The other two data sets used in this assessment are available through GEO accessions GSE83667 and GSE116341. GSE83667 consists of 58 microarray-based, *P. falciparum* transcriptomic profiles obtained from blood samples collected in Malawi (36). GSE116341 primarily consists of single-cell RNA-seq data from an *in vitro P. falciparum* blood stage strain, but also includes 28 bulk RNA-seq measurements on parasite populations, which we used for model validation (37).

FASTA and GFF files containing genome sequence and annotation information for the *P. falciparum* 3D7 strain were downloaded from plasmodb.org (38) (release 42) and used in cMonkey2 runs.

A FASTA file containing *P. falciparum* 3D7 protein sequence information across the organism's proteome was downloaded from UniProt (39) for use in identifying proteins with potential gene regulatory functions.

### Model construction

To generate our global gene regulatory network, we adapted a workflow previously used to generate EGRINs for *Halobacterium salinarum* and *E. coli* (19). First, we used the cMonkey2 biclustering tool (40,41) (version 1.2.11) to identify co-regulated genes among the transcriptomic profiles from training data (GEO accession GSE59097). The output of a cMonkey2 run is a set of biclusters, each of which contains a set of genes and a set of transcriptomic samples in which those genes show elevated expression coherence. Thus, each bicluster represents genes that are likely co-expressed as part of a common regulatory program. We then used the Inferelator, a regression analysis algorithm (20,42) (https://github.com/baliga-lab/cMonkeyNwInf) to generate a network of weighted regulator-target interactions from the collection of biclusters in each cMonkey2 run in combination with a list of *P. falciparum* transcriptional regulators. The weights of these interactions, which quantify the influence that regulators have on their target genes, were then averaged across these EGRINs to create an ensemble EGRIN. The final list of 90 300 regulator-target pairs comprising the model was determined by ranking all pairs in the ensemble EGRIN by their absolute weights, then testing model predictions on training data using various percentages of the top-ranked pairs.

In the context of EGRIN modeling, when discussing the transcriptional influence of gene products on other genes’ expression, we make a distinction between TFs and TRs. We refer to proteins that have been demonstrated to regulate target gene expression by binding to *cis*-regulatory DNA elements as TFs. We consider TRs a broader set of proteins that regulate gene expression, but not necessarily through sequence-specific DNA-binding. We make this distinction because many of the proteins presumed to have gene regulatory activity in our model have not been molecularly analyzed, and their precise modes of action as regulators remain unknown. It is a distinction we introduce to make it clear that the complement of regulatory proteins in our model is not limited to those that exert regulatory influence through sequence-specific, *cis*-regulatory DNA-binding, but also includes proteins that may influence expression via more indirect mechanisms (hence the use of the word ‘Influence’ in the term EGRIN).

### Identifying biclusters from transcriptomic data

The cMonkey2 biclustering tool identifies potential gene regulatory programs based on transcriptomic data and *a priori* biological knowledge (40,41). The tool groups genes based on their *coherence* across transcriptomic profiles; genes with expression levels that shift more in parallel across transcriptomic profiles are more likely to be grouped together by the algorithm as this suggests the genes may be under the influence of a common set of transcriptional regulators. Concomitantly, cMonkey2 identifies subsets of transcriptomic profiles (samples) in which gene sets show high coherence. This allows cMonkey2 to delineate particular experimental samples in which gene coherence is high. For example, expression in a certain regulatory pathway may only be coherent among transcriptomic profiles from samples that were treated with a certain drug.

For details on the cMonkey2 algorithm, we refer to the reader to the original paper describing the algorithm (40) and the Supplementary Information in Brooks, *et al.* (19) which describes updates to the algorithm present in the version of cMonkey2 used to construct the *Pf*EGRIN. To summarize, the algorithm uses a stochastic procedure modeled after *k*-means clustering to optimize the assignment of genes and transcriptomic samples to a pre-defined number of biclusters. First, genes and transcriptomic samples are divided into the set of biclusters using *k*-means clustering. Then, across a series of iterations, the algorithm updates the assignment of genes and samples among the biclusters based on a scoring metric that quantifies how well a gene or sample fits within a particular bicluster (analogous to determining closeness to the centroid of a *k*-means cluster). This metric incorporates measures of concordance in gene expression levels, agreement among predicted upstream gene regulators, and other data providing evidence for gene interactions such as protein-protein interaction networks. The result is a set of biclusters containing genes that show high likelihood of being co-regulated within a common gene regulatory program among samples in the bicluster.

To prepare the transcriptomic data from the GSE59097 training data set for cMonkey2 biclustering analysis, we normalized the data on a gene-by-gene basis using a *z*-score transform. The 5000 genes with the highest standard deviation across samples in the raw GSE59097 expression data were used in our runs. This cutoff was chosen to be consistent with established cMonkey2 protocols, to ensure comparability against previous EGRIN development efforts, and to avoid fitting our model to potential noise generated by transcripts with low variance while ensuring that a comprehensive set of *P. falciparum* genes remained in the model. Runs were performed using the Bicluster Sampled Coherence Metric (BSCM), which helps optimize the difference in coherence between samples within a bicluster and those not in the bicluster (43). cMonkey2 runs were performed with *de novo* binding motif detection enabled. This means that when the tool calculates the likelihood that a gene belongs within a particular bicluster, it scans the *P. falciparum* genome to determine if the gene and those in the bicluster share common upstream binding motifs and are therefore more likely to be co-regulated.

### Inferring a gene regulatory influence network from biclusters

The Inferelator tool uses elastic net regularization, which is a linear regression-based statistical modeling method, to determine which *P. falciparum* TRs regulate which target genes and to quantify the direction and strength of that regulatory influence. Given the biclusters from a cMonkey2 run and a list of TRs assumed to have regulatory influence on the organism's genes, the Inferelator generates a list of weighted TR-bicluster pairs that indicates which TRs regulate which groups of biclustered genes and to what degree. Generally speaking, this determines which TRs regulate which gene regulatory programs identified by cMonkey2. The weights of the TR-bicluster pairs quantify the influence of a TR on a gene regulatory program and are determined by the elastic net algorithm underlying the Inferelator. They represent the elastic net algorithm's best estimates for predicting the mean expression levels of genes in a bicluster from the expression levels of TRs. Our systems-level EGRIN, which is composed of relationships between TRs and target genes, is constructed from the TR-bicluster pairs: For each such pair, a regulatory relationship between the TR and each individual gene member of the bicluster is asserted in the EGRIN, and the TR-bicluster weight is used as the weight of that relationship. The resulting set of relationships can be analyzed as a node-and-edge network consisting of genes (nodes) and the directed, weighted regulatory relationships between them (edges). Using these relationships, expression levels for individual genes can be computed using only the expression values of TRs predicted to influence their expression. This can be done for any individual transcriptomic profile for which TR expression values are available. Gene expression predictions are obtained by first collecting all TR-target pairs that include the gene and have non-zero weights, and then multiplying each weight by the expression level of the TR in the pair, then summing these products across the TR-target pairs.

The Inferelator identifies regulatory relationships using correlation, and one of the challenges in developing a network using a correlation-based approach is determining the directionality of regulatory relationships. For example, if two TRs have well-correlated expression levels, this may indicate that the first regulates the second, the second regulates the first, or the regulation is reciprocal. If given temporally-resolved expression data as input, the Inferelator can address this issue using dynamic modeling of TR-target interactions. The data used here for model training were from a single time point, and dynamic modeling was therefore not applicable. However, an additional way to address the directionality issue, which was used in our approach, is by applying *de novo* upstream motif detection when using cMonkey2 to identify biclusters. Doing so adds an additional layer of biological evidence indicating that co-regulated genes in a bicluster are targets of some TR and not necessarily the reverse. Thus, while the Inferelator may find a regulatory relationship suggesting that TR_1_ targets TR_2_, it may not necessarily predict the reciprocal because upstream motif detection may assign TR_1_ to a bicluster whose overall expression profile does not correlate well with TR_2_. We therefore assume that each individual TR-target regulatory relationship identified by the Inferelator is unidirectional. Reciprocal relationships may nonetheless appear in the resulting network if the Inferelator separately identifies both elements of a reciprocal relationship between TRs.

### Determining model convergence

The cMonkey2 algorithm includes elements of randomness as it attempts to optimally group genes and samples together. For example, at the beginning of each cMonkey2 run, genes and samples are randomly divided into a specified number of biclusters. Additionally, to avoid falling into local minima, a small amount of random noise is applied to the probability matrix used to assign genes to biclusters at each iteration of the algorithm. At the end of each iteration, a random set of elements (genes or samples) is selected for bicluster re-assignment as part of the algorithm's optimization routine (19). Therefore, separate cMonkey2 runs using the same input data do not generate identical results, and an ensemble of cMonkey2 runs is needed to account for run-to-run variation and produce statistically robust results.

To determine the number of cMonkey2 runs needed to ensure robustness in our final gene regulatory model, we performed multiple cMonkey2 runs, then used separate Inferelator runs to compute TR-target weights for each individual cMonkey2 run. We determined what percentage of TR-target pairs produced by the Inferelator had average weights that shifted less than 5% when comparing average weights over the total number of cMonkey2 runs to average weights over three less than the total number of runs. If more than 95% of the averaged TR-target weights shifted less than 5%, then we concluded that additional cMonkey2 runs would not substantially change the gene regulatory network model derived from the averaged weights. We reached our target level of convergence for TR-target interaction weights after 77 cMonkey2/Inferelator runs: less than 5% of the average weights on TR-target interactions changed >5% between 74 and 77 runs (Supplementary Figures S1 and S2).

### Compiling *P. falciparum* transcription regulators

The list of TRs used as input to the Inferelator consists of known, empirically-analyzed TFs, predicted TRs reported in the literature, and predicted TRs we identified through a protein domain analysis performed on the *P. falciparum* proteome. The TR list was populated in part using the collection of regulatory genes previously compiled by Bischoff and Vaquero (17) who used Pfam Hidden Markov Model profiles (44) to identify proteins with potential transcriptional activity. Among the 202 genes they identified, their list includes the 27 genes encoding ApiAP2 proteins (45). We also included empirically-analyzed and putative TFs mentioned in a recent review (16). To further expand the list, sequences for proteins in the *P. falciparum* proteome were downloaded from UniProt (39) and then input to the InterProScan tool (46) to identify protein domains within each proteome entry. InterProScan utilizes domain information from several databases, including Pfam (44) and SMART (47), and provides textual descriptions of any domains that are found within the scanned proteins. From the InterProScan output, we selected any proteins annotated against protein domains containing the phrase ‘DNA binding’ or ‘transcription factor’. We accounted for subtle variations in these specific phrases. For example, domain annotations containing the phrase ‘dna-binding’, ‘DNA binding’ or ‘dna_binding’ were all flagged by our approach. We added the genes encoding these proteins to the existing list of TRs, accounted for overlap, then selected those genes that were present in the list of 5000 used for our cMonkey2 runs. This final list of genes was used as input to each of the Inferelator runs we performed to generate our ensemble of EGRIN models.

### Model evaluation

Evaluations using the three transcriptomic validation data sets compared measured and model-predicted mean gene expression across the biclusters generated from our 77 cMonkey2 runs. For all three validation data sets, the model was used to predict expression values for each gene in a bicluster using the measured expression levels of the TRs regulating those genes and the weight of those interactions in our model. For each bicluster tested, the mean model-predicted expression of its genes was computed and compared to the mean measured expression from the validation data set. To ensure the comparisons were valid, we normalized the validation data using the same method applied to the training data used to create the model (gene-by-gene z-score transform). To quantify model accuracy, we calculated root-mean-square errors (RMSEs) between the predicted and measured values across samples in each bicluster. In some cases, due to missing values in samples within the validation sets, we were not able to compare the full complement of predicted and measured values for each sample. Therefore, for a bicluster to be included in this analysis, we required that more than half of the samples in the validation data set contain the full complement of predicted-to-measured gene expression comparisons. An archive of R scripts and data objects that allow a user to reproduce model-based, quantitative predictions reported here is included as Supplementary Data.

For evaluations that assessed overlap between model-predicted targets of ApiAP2 TFs and targets predicted based on empirically-derived binding sequence motifs, we used the ‘TF Binding Site Evidence’ page on plasmodb.org to retrieve lists of genes that are targeted by ApiAP2 TFs based on the presence of TF-specific upstream binding motifs. We used the page's default parameters to set the size of the upstream region to scan (1000 bp), the minimum number of motifs per gene (1 motif) and the minimum confidence level for a match to the motif (*P*-value ≤ 1e–4). We then compared the retrieved lists to the targets of ApiAP2 TFs predicted by our model. For each ApiAP2 TF evaluated, a hypergeometric enrichment test was used to quantify the overlap between motif-based and model-based target predictions.

### Identifying correlates of artemisinin resistance

Hypergeometric enrichment tests were used to identify biclusters that were either significantly enriched for samples with AR infections or significantly enriched for samples with AS infections. In accordance with previous criteria used to analyze the training data used to build our model, we classified samples with parasite clearance half-lives greater than or equal to 5 h as AR (30). Samples with lower clearance times were classified as AS. After identifying biclusters that showed over-representation of either the AR or AS group, we functionally profiled the genes in the biclusters by determining their enrichment for members of gene sets collected from the Gene Ontology (48,49), the Malaria Parasite Metabolic Pathways (MPMP) resource (50,51), and data sets associated with stage-specific gene expression at different points in the parasite life cycle (52–59).

## RESULTS

### Gene regulatory network model construction

To generate a robust *P. falciparum* EGRIN, we adapted a workflow previously used to create EGRINs for *H. salinarum* and *E. coli* (19). As illustrated in Figure 1, we first used the biclustering tool cMonkey2 (40,41) to identify genes and transcriptomic samples that group together based on gene expression coherence. These biclusters were computed based on a large *P. falciparum* transcriptomic data set (GEO accession GSE59097) and the genomic sequence of the organism. We then used the Inferelator tool (20,42) to generate a network of weighted TR-target interactions based on coherence between TRs and biclustered genes. The list of TRs used for this step was compiled from known, empirically-analyzed TFs (16), putative regulators compiled via computational methods in a previous study (17), and an analysis we performed using InterProScan (46) that identified additional proteins with potential regulatory activity. For semantic clarity, we reserve the TF term for proteins that have been shown to bind to *cis*-regulatory DNA elements; TRs are a broader class of proteins including TFs whose regulatory activity is not necessarily limited to sequence-specific binding. In the following sections, we detail results from each step in our workflow which ultimately culminated in our system-level gene regulatory network.

### Generating biclusters

**Figure 2.**
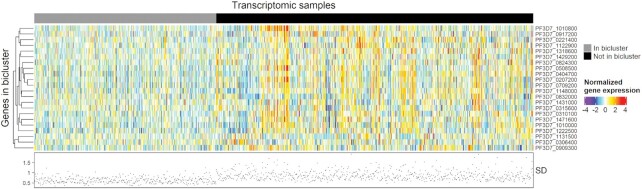
Example of cMonkey2 biclustering. Heatmap colors indicate normalized expression values for genes in an example bicluster across 380 transcriptomic samples within the bicluster (gray annotation bar) and 663 samples not in the bicluster (black annotation bar). Scatterplot below heatmap indicates standard deviation (SD) of expression across genes for each sample. Samples in the bicluster show lower SD (more coherence) compared to the samples not in the bicluster.

The primary product of a cMonkey2 run is a set of biclusters, each of which contains a set of genes and a set of samples whose transcriptomic profiles show elevated coherence among those genes. Put another way, there are a set of transcripts that increase and decrease together, within a set of samples. These biclusters identify genes that may be part of a common regulatory program and thus, form the foundation of EGRIN models (Figure 2). The difference between coherent gene expression among samples within a bicluster and the remaining samples in the data set can be visualized by normalized gene expression (Figure 2, top) or by examining the standard deviation between transcript levels of genes within the bicluster (Figure 2, bottom). Here, we have used the biclusters output by cMonkey2 to (i) group *P. falciparum* genes into distinct regulatory programs that are then associated with TRs to build our EGRIN model, and (ii) identify genes that are associated with artemisinin resistance or sensitivity by identifying biclusters whose sample membership showed enrichment for either artemisinin resistant or artemisinin sensitive samples.

Because cMonkey2 involves stochasticity in the biclustering initialization process as well as each iteration of the optimization process, cMonkey2 runs are not deterministic. Thus, the biclusters generated between runs are not identical, and an ensemble of cMonkey2 runs is needed to ensure the results are statistically robust. To determine the required number of cMonkey2 runs that would result in a robust *Pf*EGRIN, we performed runs until 95% of the TR-target weights generated by the Inferelator from each run showed convergence based on their average values across runs. We found that our model showed convergence after 77 cMonkey2 runs (Supplementary Figures S1, S2).

### Identifying candidate transcription regulators

Building an EGRIN model requires both cMonkey2 runs and a list of TRs as input to the Inferelator (Figure 1). Our compiled list of TRs includes the ApiAP2 proteins, a previously compiled list of computationally-predicted transcription-associated proteins (17), empirically-analyzed and additional candidate TRs (16), and additional proteins that we identified as having potential regulatory activity (Supplementary Table S1). We identified this latter group of proteins in the interest of being inclusive in our TR list, given that much of the gene regulatory landscape of *P. falciparum* remains uncharacterized. Furthermore, whereas our network-construction methodology can optimize the model by removing TRs with little or no regulatory influence, it cannot add TRs as part of this optimization process. Therefore, we aimed to compile an inclusive list of potential TRs so that we did not exclude genes with critical regulatory roles. Using the InterProScan tool, we identified proteins in the *P. falciparum* proteome containing protein domains that indicate potential gene regulatory functions. Out of the full *P. falciparum* proteome, 4996 proteins were annotated with at least one protein domain. Of these, 105 were annotated with domains that suggested a potential role as a TR, 92 of which were in the list of 5000 genes used as input to the cMonkey2 runs (see *Identifying biclusters from transcriptomic data*). Twenty-four of the 92 were in the list of TRs that we had already compiled from literature sources. Thus, through this analysis, 68 novel candidate TRs were added to the previously compiled list of TRs, bringing the number of TRs used to train our EGRIN to 258.

### Generating and quantifying the gene regulatory influence network

Using a set of TRs and the biclusters from a cMonkey2 run, the Inferelator uses elastic net regularization to produce a list of TR-bicluster pairs with weights that quantify the influence of the TR on the genes in the bicluster (Figure 1). These weights are then assigned to each gene in that bicluster to generate a network consisting of weighted edges that link TR-target gene pairs. For each of our cMonkey2 runs, we performed a separate Inferelator run to generate a TR-target network, then aggregated the results from the Inferelator runs into an ensemble network as described previously (19). Using an input set of TR expression values in the ensemble network, we can then predict the expression level of an individual gene by identifying which TRs regulate the gene, then computing the dot product of the weights on the TRs regulating the gene and the TRs’ expression levels.

In accordance with previous EGRIN modeling efforts, we initially used the top 100 000 TR-target pairs with the highest absolute weights in our ensemble network for our *Pf*EGRIN model. To determine if this cutoff would produce the most predictive model, we performed an optimization analysis that compared the predictive capabilities of models consisting of different numbers of top-ranked TR-target pairs. This analysis assessed the accuracy of model predictions on mean bicluster gene expression across the 38 500 biclusters generated by our 77 cMonkey2 runs. We found that a model consisting of 7% (90 300) of the top-ranked TR-target interactions from the ensemble gene regulatory network provided the best fit to the training data based on mean RMSE values (Supplementary Figure S3). The final *Pf*EGRIN model, therefore, consists of these 90 300 TR-target pairs. An R data object file listing these interactions and their weights is provided in the Supplementary Data.

### Gene regulatory network characteristics

The final *Pf*EGRIN model consists of 5000 genes (nodes) and 90 300 TR-target interactions (weighted edges) (Table 1, Supplementary Figure S4). Positive regulation of target genes by TRs dominates (86%) the network. All 5000 genes, including the 258 TRs, are predicted to be regulated by at least one TR. Five TRs had no influence on the expression of other genes in the model but were regulated by other TRs. These included two TRs from the list compiled by Bischoff and Vaquero (17) (PF3D7_0215700 and PF3D7_1353500) and three TRs included based on our InterProScan analysis (PF3D7_1233000, PF3D7_1235400, and PF3D7_1312400), none of which are members of the ApiAP2 family. Thus, the model includes 253 TRs that influence the expression of target genes.

**Table 1.** Connectivity metrics from the TR-target regulatory network

		Number of interactions	
Number of genes	Number of TRs	+	−
5000	258	77 639	12 661

**Figure 3.**
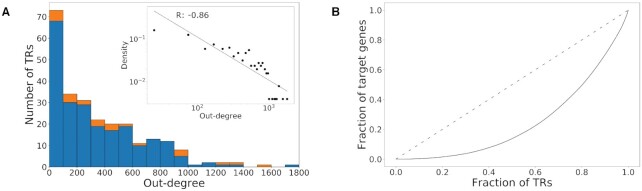
Outgoing connectivity of gene regulatory network. (**A**) Histogram of out-degree distribution. Orange shading in stacked bar plot indicates number of ApiAP2 TFs in a bin; blue shading indicates number of other TRs. Inset: Probability density of TRs and their out-degrees on a log–log scale with regression line. R is the Pearson correlation coefficient. (**B**) Lorenz curve of TRs and their target genes. Dashed line indicates line of equality.

To investigate the topology of the regulatory network, we performed network analyses focused on the outgoing connectivity (out-degree) of TR nodes. The majority of TRs regulate a small number of target genes, while few TRs regulate a relatively large number of target genes (Figure 3A). Comparing the probability density of TRs and their out-degrees on a log-log scale revealed a linear relationship (Pearson correlation coefficient = –0.86, *P*-value = 3.6e–9), indicating that the network degree distribution follows a power-law (Figure 3A, inset). This suggests a scale-free topology, which is a general characteristic of many networks in nature and society (60–62). For a scale-free network, the probability of a node having an out-degree of *k* follows the power-law function

$$\begin{equation*}p\left( k \right) \sim {k^{ - \gamma }}\end{equation*}$$

where γ is the power law scaling exponent. The scaling exponent of our *P. falciparum* gene regulatory network is 1.01, which is smaller than empirically-derived exponents obtained from other eukaryotic model organisms including *C. elegans* (4.12), *D. melanogaster* (3.04), *S. cerevisiae* (2.0) and *A. thaliana* (1.73) (63). The scale-free connectivity suggests that gene regulation is performed by a relatively low number of highly influential TRs. We ranked the TRs based on their out-degrees and plotted the cumulative proportion of TRs against the cumulative proportion of the corresponding target genes (Lorenz curve (64); Figure 3B). The deviation of the curve from the line of equality again demonstrates that a small fraction of TRs regulates a significant number of target genes in the *Pf*EGRIN model.

As part of these network analyses, we also examined whether the approach used to compile our TR list was overly inclusive and resulted in an excess of TRs with low connectivity. If, for example, the TRs added through our InterProScan analysis contained proteins with substantially less impact on gene regulation, we would expect that the out-degree distribution of that set of TRs to skew toward lower out-degrees. However, we found that the distribution of out-degrees among ApiAP2s proteins, non-ApiAP2 TRs compiled previously (17), and the additional TRs we identified in our InterProScan analysis did not differ significantly (Kruskal–Wallis test, *P*-value = 0.32, Supplementary Figure S5). These results indicate that these TR categories influence the overall network according to a similar pattern of connectivity and suggest that our approach for compiling TRs was appropriately inclusive.

The majority of TRs (249/258) included in the EGRIN model influence <1000 genes. There are nine TRs that each influence >1000 genes with PF3D7_0407600 having the highest out-degree (1791). Together, these nine TRs influence 87.8% of the target genes in the model (4390). To assess their functional roles, we tested each TR’s set of regulatory targets for enrichment of functional gene sets from GO, the MPMP resource, and sets associated with specific parasite life cycle stages. The median number of sets significantly enriched among these ‘hub’ TRs was 38 (range: 23–73). The TR with the highest number of targets, PF3D7_0407600, showed the highest number of enriched sets. For all nine TRs, their gene targets were significantly enriched for GO:*antigenic variation*, GO:*host cell plasma membrane*, MPMP:*Interactions between modified host cell membrane and endothelial cell*, MPMP:*Rosette formation between normal and infected RBC*, and MPMP:*Structure of telomere and subtelomeric regions* (hypergeometric test, FDR-corrected *P*-value < 0.05). These results, which link the predicted ‘hub’ TRs in our model to antigenic variation and host-parasite cell interactions, are consistent with the critical functions of ring-stage parasites such as those whose transcriptomes were used to build our model (30).

The TR with the highest number of regulatory targets, PF3D7_0407600, is an uncharacterized gene that has received little attention in the *P. falciparum* gene regulation literature. It is included in our list of compiled TRs based on its presence in the TR list compiled by Bischoff and Vaquero (17) whose analyses identified C2H2-type zinc finger domains in this protein. The TR’s high predicted out-degree in our model suggests that the gene's protein product may play a critical regulatory role in blood-stage parasite infections, and our functional gene set enrichment analysis of the targets of PF3D7_0407600 suggests it may regulate a variety of biological functions. Gene sets with the highest enrichment scores include those associated with the parasite's gametocyte and late-ring stages as well as protein phosphorylation and host-parasite cell interactions.

The TR with the second-highest number of regulatory targets is PF3D7_1007700 (PfAP2-I). This gene encodes an ApiAP2 protein that plays a important role in erythrocyte invasion (11). Our model-predicted targets of PfAP2-I enriched for 23 functional gene sets, including sets associated with antigenic variation, invasion, and host cell remodeling. These results support previous findings that PfAP2-I plays a critical regulatory role in the parasite's blood stage development.

The full list of significantly enriched gene sets for all nine ‘hub’ TRs with more than 1000 gene targets is available in Supplementary Table S2. For some of these TRs, the mechanism underlying their influence on gene expression remains unknown. Thus, we have also included the protein domains identified by our InterProScan for each of these nine TRs in Supplementary Table S2 as they can suggest potential mechanisms of regulatory action.

### Model predictions on gene expression

We evaluated the capability of the *Pf*EGRIN to predict transcriptomic gene expression levels using three validation data sets not used to train the model. These transcriptomic data sets were selected so that the model would be tested against data obtained from a variety of research groups, using a variety of measurement modalities, and from blood-stage parasite populations originating from different regions (Table 2). This allowed us to assess the model's predictive capabilities across geographic locations, across transcriptomic platforms/processing pipelines, and across parasite populations cultured from clinical isolates or from *in vitro* laboratory stocks.

**Table 2.** Descriptions of transcriptomic validation data sets used to evaluate the *Pf*EGRIN model's predictive performance and summary statistics for each test. Summary statistics for root mean square error (RMSE) and correlation coefficients are presented as median values followed by the inter-quartile range

	Validation test 1	Validation test 2	Validation test 3
*GEO accession*	GSE59098	GSE83667	GSE116341
*Associated publication*	Mok *et al.* 2015 (30)	Milner *et al.* 2012 (36)	Ngara *et al.* 2018 (37)
*Number of samples*	110	58	28
*Source of parasites*	Clinical isolates (Cambodia)	Clinical isolates (Malawi)	*In vitro* strain *Pf*3D7
*Measurement technique*	Microarray	Microarray	RNA-seq
*Biclusters evaluated*	10 462	6341	10 078
*Unique genes evaluated*	4515	3985	4473
*RMSE for* Pf*EGRIN, training data*	0.26 (0.21–0.44)	0.28 (0.21–0.44)	0.26 (0.21–0.44)
*RMSE for* Pf*EGRIN, validation data*	0.29 (0.22–0.44)	0.29 (0.22–0.38)	0.33 (0.25–0.44)
*RMSE for random model, validation data*	0.73 (0.65–0.81)	0.72 (0.64–0.81)	0.53 (0.42–0.68)
*Correlation coefficient for* Pf*EGRIN, training data*	0.97 (0.96–0.98)	0.98 (0.97–0.98)	0.97 (0.96–0.98)
*Correlation coefficient for* Pf*EGRIN, validation data*	0.95 (0.92–0.97)	0.94 (0.90–0.98)	0.85 (0.69–0.92)
*Correlation coefficient for random model, validation data*	0.16 (0.03–0.32)	0.18 (0.03–0.34)	0.13 (-0.03–0.29)

**Figure 4.**
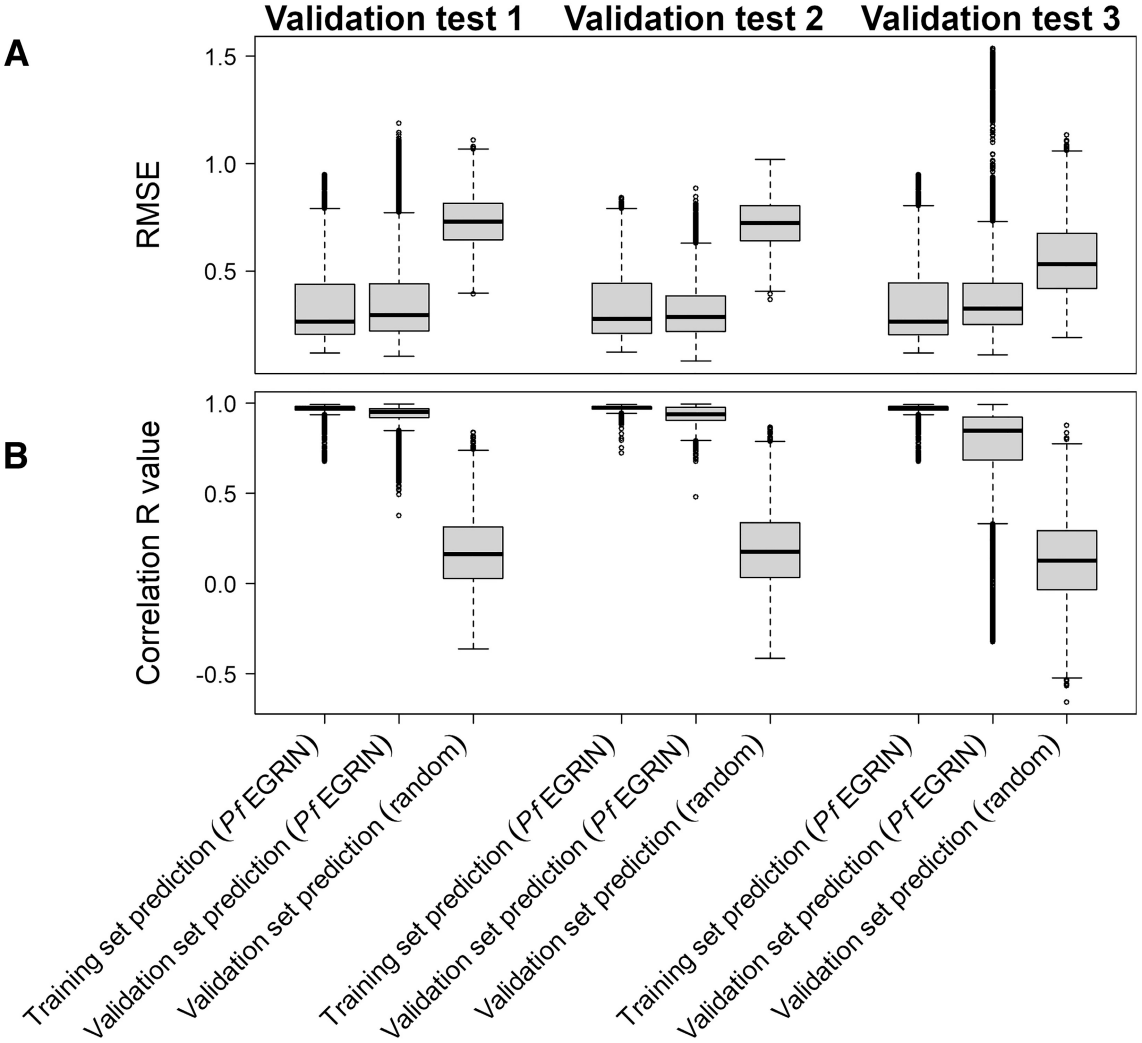
*Pf*EGRIN model predictive performance. (**A**) Distributions of root-mean-squared error (RMSE) in model-based gene expression predictions across cMonkey2 biclusters generated in building the model. For each of our three validation tests, we used the model to predict mean expression among biclustered genes across biclustered samples from training data as well as samples from validation data. For comparison, we also made predictions on the validation data using a model that randomly selects values from a normal distribution. (**B**) Pearson correlation coefficients (*R*) for the same predictions in (A).

**Figure 5.**
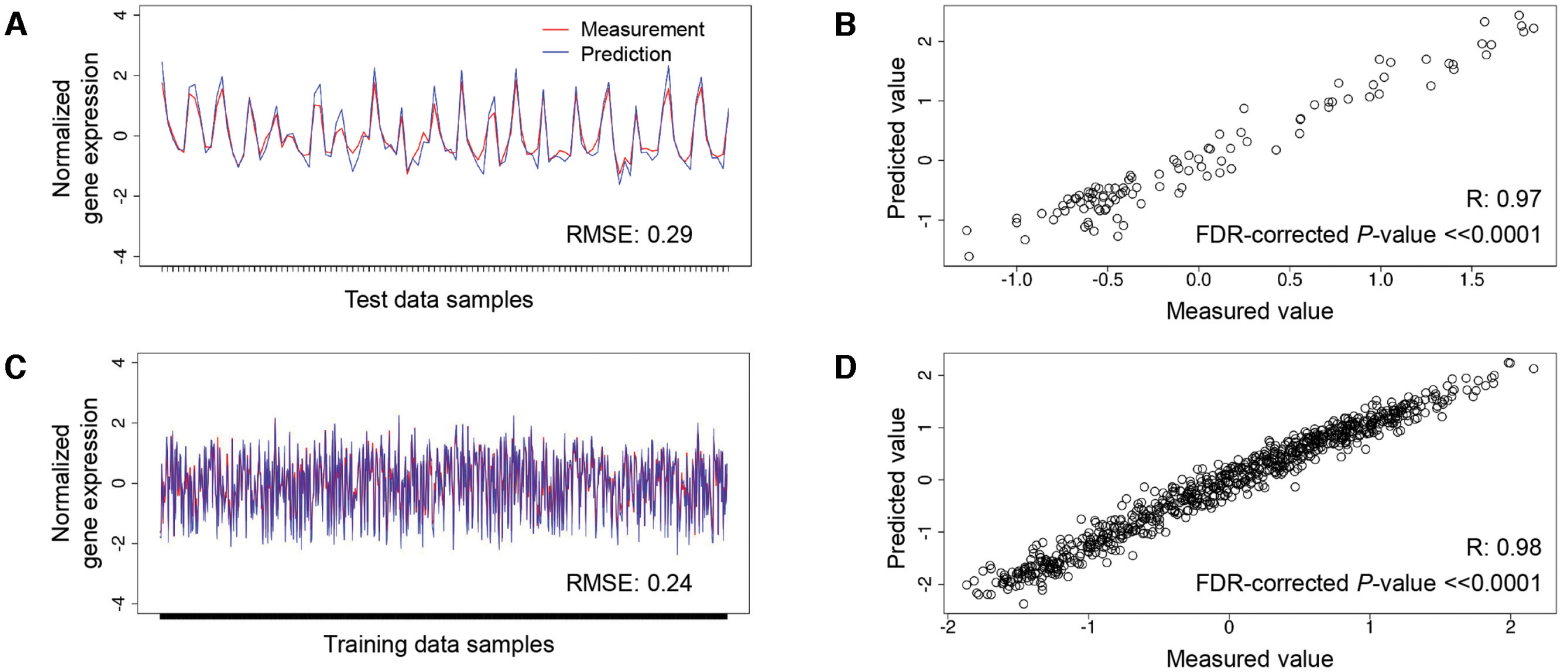
Representative predictions made by the *P. falciparum* EGRIN model. (**A**): Measured gene expression levels from the validation test 1 data set (red) compared to model predictions (blue) for genes in a representative cMonkey2 bicluster. Model accuracy in predicting expression levels for genes in this bicluster was similar to the model's overall prediction performance for the validation set. (**B**) Scatterplot showing correlation between measured and predicted expression values in (A). *R* is the Pearson correlation coefficient. (**C** and **D**): Same as in (A) and (B), with measured and predicted expression levels for samples in the GSE59097 training set contained in the bicluster.

Overall, we found that predictions on validation data were only slightly less accurate than predictions on the training data (Table 2). We evaluated the distribution of RMSE values (Figure 4A) and correlation R values (Figure 4B) when using the *Pf*EGRIN model to predict expression values for biclustered genes on samples from the training set (those within a bicluster) and on all samples from the validation set, or when using a model that predicts expression based on random value selection from the normal distribution. Figure 5 shows sample-by-sample model predictions on validation data set 1 (GEO accession GSE59098) for an example bicluster in which predictive accuracy on the validation set was equal to the median of the model's overall performance and is representative of all biclusters tested. Figure 5A compares, across samples in validation data set 1, the measured mean normalized gene expression values for genes in the bicluster against model-predicted values. Figure 5B shows the correlation between these values. The RMSE and correlation coefficients for these predictions on validation data set 1 are similar to the performance of the model on the training data shown in Figure 5C and D.

The median RMSE values and correlation coefficients for model-based predictions on data from samples used to train the model were similar across our three validation tests (Table 2, Figure 4). Although similar, these values are not equivalent. This is because some biclusters did not meet our criteria for inclusion in a validation test due to missing gene expression values (see Materials and Methods) and the three validation test sets contain different complements of missing data values. Therefore, the different validation tests excluded different members of the 38 500 biclusters generated from our cMonkey2 runs. Consequently, the total number of unique genes assessed in our evaluations also differed across validation tests.

Median RMSE scores were similar for *Pf*EGRIN predictions over the three validation tests and were significantly lower than those based on random predictions (*P* < 2.2e–16 for each validation set; paired Wilcoxon rank-sum test). Median correlation coefficients between *Pf*EGRIN-predicted and measured expression values were high (≥0.85) across the training and validation data sets (Table 2, Figure 4B).

### Model-based versus motif-based ApiAP2 target prediction

We next evaluated the ability of the *Pf*EGRIN to identify targets of ApiAP2 TFs, which regulate gene expression throughout different stages of the *P. falciparum* life cycle. We assessed the agreement between the model-predicted gene targets of ApiAP2 TFs and targets predicted based on empirically-determined upstream sequence motifs specifically recognized by those ApiAP2 TFs (9). Lists of predicted targets for each ApiAP2 based on upstream binding motifs were downloaded from plasmodb.org and compared to targets predicted by our model. Out of 18 ApiAP2 TFs compared, four showed significant overlap between motif-based and model-based target lists (hypergeometric test, FDR-corrected *P*-value < 0.05). In order of significance, these ApiAP2s were PF3D7_0802100, PF3D7_1456000, PF3D7_0604100 and PF3D7_1305200. Notably, mRNA abundances of the top three TFs have similar dynamics during the parasite's intraerythrocytic developmental cycle (IDC), peaking in the early-mid schizont stage (9).

### Incoherent expression is associated with artemisinin resistance

**Figure 6.**
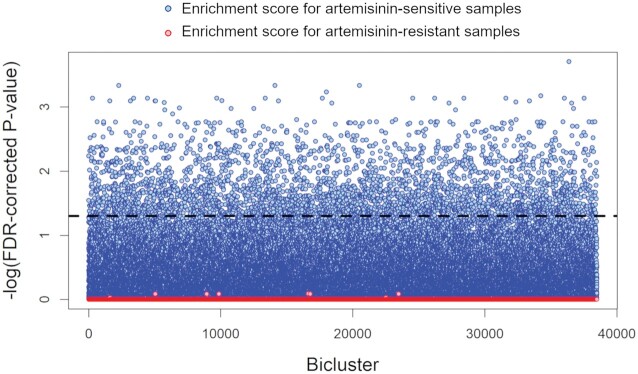
Hypergeometric enrichment scores for artemisinin-resistant and artemisinin-sensitive samples among all biclusters generated in constructing the *Pf*EGRIN. Dashed line indicates –log_10_ of an FDR-corrected *P*-value of 0.05.

Because the biclusters used to develop the *Pf*EGRIN were generated using transcriptomes from parasites with various levels of artemisinin sensitivity, we reasoned that we might be able to identify biclusters that exhibit concordance selectively in samples showing AR. However, across all 38 500 biclusters generated, no FDR-corrected *P*-values for AR sample enrichment fell below 0.81. In contrast, 3202 biclusters (8%) met an FDR-corrected *P-*value cutoff of 0.05 for enrichment of AS samples (Figure 6). This suggested that AR is primarily associated with *in*coherence rather than coherence among gene regulatory programs.

### Correlates of artemisinin sensitivity

Considering gene expression was incoherent among AR samples, we identified the genes and biological functions most strongly associated with the AS biclusters. We first selected the most high-confidence AS biclusters by identifying those with FDR-corrected enrichment *P-*values <0.01. We then tabulated and ranked the number of times each gene appeared in one of these 710 highly enriched AS biclusters. We identified 111 genes that appeared more often than would be expected by chance (hypergeometric test, FDR-corrected *P*-values < 0.05) (Supplementary Table S3). Among this set are 35 *var*, 27 *rifin*, 8 putative ribosomal component, 2 ApiAP2 TFs, 2 putative proteasome subunit and 2 putative dynein heavy chain genes. Interestingly, we found that the highest-ranking genes were members of the parasite's *var* gene family, which encode a group of immunovariant proteins involved in antigenic variation and erythrocyte adhesion at sites of vascular infection. They are a central component of the parasite's strategy for evading immune clearance by the host, and a body of evidence suggests they are under the control of epigenetic pathways (reviewed in (65)); however, the interpretation of this result based on microarray data is confounded by the high sequence complexity of genes in the *var* and *rifin* families (see Discussion).

**Figure 7.**
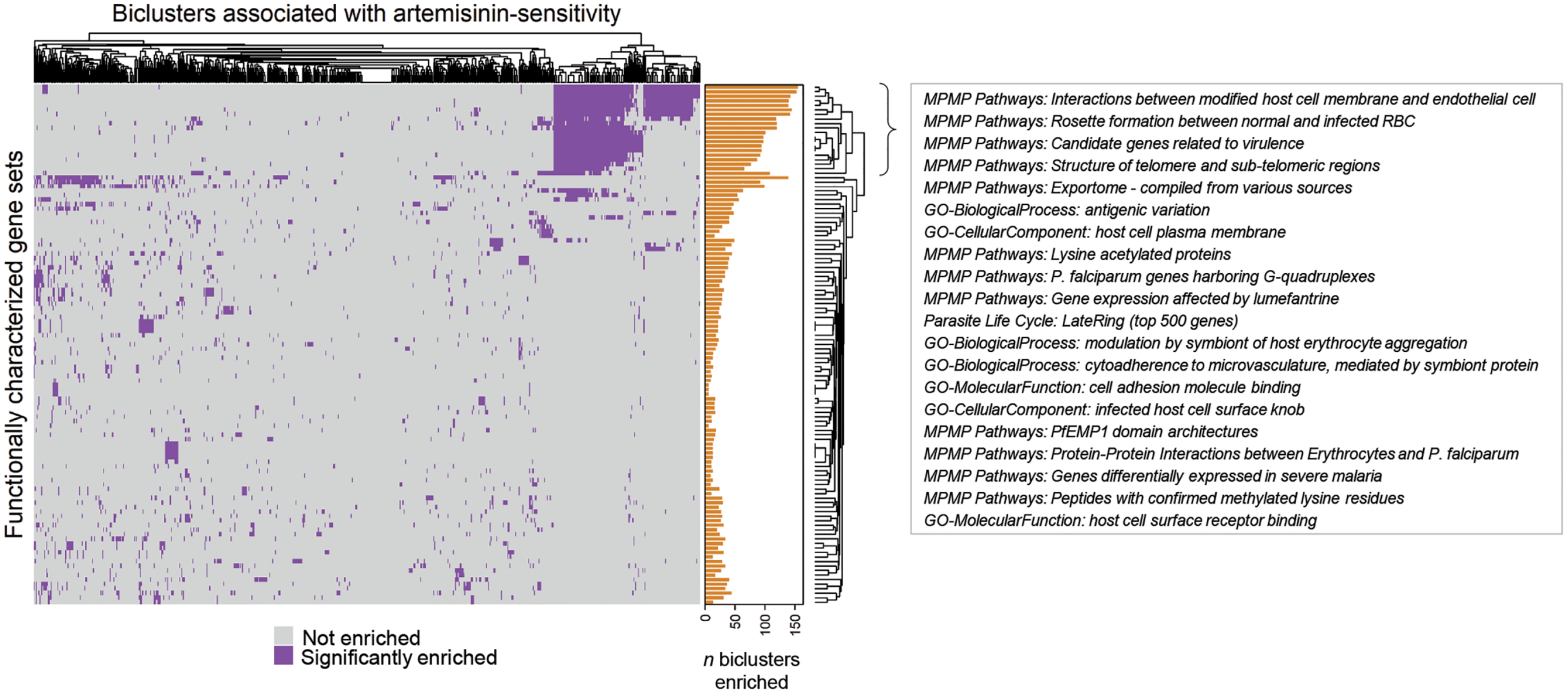
Heatmap showing clusters of functional gene sets among the 710 biclusters most highly enriched for artemisinin-sensitive samples. Annotation bar plot indicates the total number of biclusters that were enriched for a given gene set. The gene sets in the top-most clade in the first branch of the row dendrogram are shown in the text box.

To assess the broader landscape of biological functions associated with AS biclusters, we assessed their enrichment for the same functional gene sets used to functionally profile ‘hub’ TRs in our *Pf*EGRIN, as described above. We identified 115 gene sets for which AS biclusters were significantly enriched (Figure 7, hypergeometric test, *P* < 0.05). For this analysis we initially defined enrichment scores with FDR-corrected *P*-values <0.05 as significant. However, a substantial fraction of AS biclusters (68%) exhibited no enrichment across gene sets using this criterion and thus could not be functionally profiled. To help illuminate the functional roles of these biclusters, we relaxed the criterion and defined enrichment scores with uncorrected *P*-values <0.05 as significant. To ensure that we only included gene sets that showed enrichment more often than would be expected by chance, we determined the null distributions of the gene sets for our biclusters by randomly sampling 710 biclusters from our complete set of 38 500 and performing functional gene set enrichment tests on them. This process was repeated 10 000 times to build null distributions for each functional gene set. For our functional enrichment analysis, we only included those gene sets that appeared significantly more often in the 710 AS biclusters as compared to occurrences in their null distribution (95th percentile or higher).

The gene sets associated with the most prominent of several clusters in Figure 7’s heatmap are delineated by the top-most clade in the first branch of the row dendrogram. Twenty gene sets in this clade have a relatively high occurrence among AS biclusters; 41% of the biclusters are enriched in at least one of these sets. Many of these sets include genes involved in antigenic variation, the export of cell adhesion proteins to the host cell membrane by the parasite, as well as erythrocyte-erythrocyte and erythrocyte-endothelium adherence. Each of these processes has been shown to be regulated epigenetically (66) and are central features of the parasite's ability to evade immune system clearance. However, no single functional profile dominates among the AS biclusters. There are many additional functional gene sets associated with them, including - but not limited to - sets related to the cell cycle, metabolism, ribosome structural components, and ion transport. Thus, AS appears associated with coherence across a variety of biological functions, and by extension AR is associated with incoherence in these functions.

## DISCUSSION

The EGRIN model of *P. falciparum* gene regulation described here is capable of making systems-level, quantitative predictions on samples from three separate validation data sets with a level of accuracy similar to those reported in previous EGRIN development efforts that were experimentally validated (20). We expect the model will serve the research community as a valuable hypothesis-generation tool for investigations into *P. falciparum's* gene regulatory biology. The model's predictive performance across validation data sets suggests that it is applicable to a variety of *P. falciparum* populations, including those clinically isolated from different geographic regions and from *in vitro* laboratory strains. Thus, it provides a critical step towards using transcriptomic data collected from field-isolated malaria parasites to predict critical parasite phenotypes and could contribute to tracking the emergence of drug resistance. Despite the heterogeneity in parasite origins and transcriptomic measurement techniques among the three validation data sets, the predictive performance of the model was generally consistent across validation tests. Even when tested against the third validation set, which differed from the training set in that it was obtained from an *in vitro* laboratory strain as opposed to clinical isolates and consists of data from RNA-seq as opposed to microarray measurements, the model showed only a modest decrease in predictive performance (Figure 4). This decrease may be due to several factors. Field isolates likely explore a larger gene expression space than laboratory strains, and because our model was trained on field isolates from a variety of locations, its predictive power across samples from laboratory strains may be reduced compared to samples collected in the field. Additionally, expression profiling using RNA-seq provides for a higher dynamic range than microarray experiments; the differences in accuracy and resolution between these modalities may contribute to the reduced predictive performance on RNA-seq samples, especially for genes with lower expression levels.

Comparisons between model-based predictions of ApiAP2 TF targets and motif-based targets showed significant agreement for four out of 18 ApiAP2s tested. For the three TFs showing the highest level of agreement, mRNA abundances all peak during a relatively tight time interval during the early-mid schizont stage of the parasite's intraerythrocytic developmental cycle (IDC) (9). Because our model uses gene expression coherence, as opposed to abundance, to identify co-regulated genes, the results of this analysis may be explained by timing differences between peak coherence of TF targets and peak abundance of TFs regulating those targets. Based on gene expression levels, the parasite populations used to train our model were found to be predominantly in the ring stage and expression levels of the top three ApiAP2s that showed significant agreement are relatively low (30). It may be that as the parasite initiates gene regulatory programs that transition the organism from stage to stage, ApiAP2-specific binding motifs become accessible and expression coherence among genes possessing those motifs is initially high. Then, as downstream regulatory mechanisms are activated by these programs, coherence decreases among those genes. In such a scenario, agreement between model-based and motif-based TF targets would be highest among TFs whose expression peaks in more downstream IDC stages. For TFs with peak expression during the ring stage, the model's predicted targets would be influenced by the accumulation of systems-level changes in the parasite's regulatory network brought on by increased TF expression, including feedback mechanisms influencing the expression of those TFs’ target genes. Thus, for our model-based versus motif-based target analysis, we do not necessarily expect agreement to be high among TFs with peak expression during the ring stage. We would instead expect agreement to be highest among TFs whose targets are newly transcriptionally active.

While the vast majority of predicted *P. falciparum* TRs require experimental validation, the model generated here can guide *P. falciparum* molecular biology research by identifying which proteins are likely to function as TRs, their predicted targets, binding sites and biological processes with which they associate. For example, by determining which TRs' target genes are over-represented in biclusters enriched for AS samples, we have used the model to create a ranked list of putative TRs that appear to be associated with artemisinin sensitivity. We note that the TRs used in our model likely do not circumscribe the complete list of *P. falciparum* TRs. Nonetheless, the accuracy of our model indicates that the TR list we compiled is sufficient to generate a model with significant predictive power. While the model can potentially generate insights into *P. falciparum's* gene regulatory programs, we may find that subsequent versions of the model with a refined list of TRs improves prediction accuracy and influences other model characteristics such as TR-target network topology.

Our network analysis of the EGRIN model suggests that the degree distribution of the *P. falciparum* gene regulatory network follows a power-law with a scaling exponent of 1.01, which is lower than those observed in networks derived empirically from studies on other model organisms. Previous research has found that for networks with scaling exponents <2, a relatively smaller set of nodes are required to control the entire network (67). Therefore, one interpretation of our results is that control of the *P. falciparum* gene regulatory network may be tighter compared to other organisms. However, additional post-transcriptional control of TRs, not incorporated into this model, likely also influences gene expression. Moreover, while our methodology quantifies the influence of each TR on all genes in the network, and the algorithm penalizes low-influence effects to exclude them from the network, the presence of relatively minor contributions of TRs to target gene expression may result in inflated TR out-degrees compared to the true physical biological network. This over-estimated connectivity could potentially result in lower power-law scaling exponents. Ultimately, a more comprehensive understanding of *P. falciparum* regulatory biology based on empirical studies is needed to validate the network characteristics derived from our model and to provide additional data to incorporate into future *Pf*EGRIN versions. We also note that the network characteristics derived from our model are based on data from blood stage parasites only. Whether these characteristics generalize across the various life cycle stages of the parasite requires further investigation. Moving forward, we are interested in directly comparing *P. falciparum's* regulatory network characteristics to those of other organisms to illuminate the organism's transcriptional control strategy and determine if and how the networks of obligate parasites such as *P. falciparum* differ from non-obligate organisms.

Overall, our efforts to find correlates of artemisinin resistance led to the discovery that resistance is linked to broad, increased transcriptional incoherence across a wide range of cellular processes (Figure 7). By comparison, hallmark constituents of the parasite's epigenetically controlled mechanisms linked to virulence or the ability to persist under immune pressure (34,68) appeared highly coherent in sensitive populations (Supplementary Table S3, Figure 7). From these results, we hypothesize that artemisinin resistance may be an emergent property of parasite populations that have higher capacities for exploring diverse phenotypic states during infection. Parasite populations with more diversity in their gene regulatory programs may be better equipped to evade immune system clearance and establish infection sites, resulting in a higher parasite burden that requires more time to clear following artemisinin treatment. This observation also links immune evasion mechanisms with the capacity to circumvent drug pressure. This linkage, if substantiated, has critical consequences for malaria eradication, where current strategies include the use of mass drug administration, particularly in areas of high transmission that are populated with diverse, pre-existing anti-PfEMP1 immunity.

Bet-hedging, wherein organisms incur a fitness cost by generating phenotypically-diverse sub-populations that allow the overall population to persist in stressful conditions, is a proposed survival strategy of *P. falciparum* (33,34). Our findings are consistent with parasite heterogeneity and bet-hedging being associated with survival in the presence of artemisinin. This is in contrast with the traditional view of the development of drug resistance, where it is presumed that a ‘sweep’ of drug-sensitive parasites decreases genetic diversity within the population, yielding a relatively genetically homogenous set of parasites. A bet-hedging strategy has no requirement for genetic diversity. Rather, phenotypic and epigenetic heterogeneity, revealed by transcript incoherence present before drug treatment, enables a subset of the population to survive drug treatment. We hypothesize that the appearance of artemisinin resistance within a parasite infection may reflect a differentiation process analogous to the epigenetically-controlled transition from asexual to sexual parasite forms (68), allowing parasite populations to explore various phenotypic states. This process may be an inherent mechanism used by the parasite to keep blood stage populations sufficiently diverse so they persist under ongoing immune system stress.

Much of our current understanding of artemisinin resistance on a cellular level implicates macromolecular, global cellular processes. This is consistent with the notion that an assemblage of altered cellular states might precede resistance. These states include global processes such as production of phosphatidylinositol 3-phosphate containing vesicles (28), oxidative stress (29), the unfolded protein response (30) and hemoglobin endocytosis (31). Therefore, it would be reasonable to hypothesize that a systematic response within the parasite could modulate each of these cellular processes, leading to a diversity of cellular states, resulting in decreased artemisinin sensitivity. This is consistent with the extensive research on the function of the Kelch13 protein, which has been linked to some artemisinin resistant strains (26,27), because various cellular pathways have been implicated in order to explain how Kelch13 contributes to resistance (31,32). Within the context of the parasite's bet-hedging mechanisms, investigations into whether Kelch13 modulates the phenotypic diversity of clonal parasite populations, and whether mutations in the protein increase that diversity, would be warranted.

Our results illustrate a key advantage in using systems-level perspectives to understand infectious disease processes such as drug resistance. The holistic, integrated perspective we have applied here has illuminated widespread differences in gene expression coherence between AS and AR infections. Based on our functional profiling results (Figure 7), there is a clear collection of biological functions associated with AS biclusters that involve genes contributing to antigenic variation and erythrocyte-host interactions. These associations are driven largely (but not exclusively) by the over-representation of *var* and *rifin* genes among a subset of the AS biclusters. We acknowledge that interpreting microarray results that focus on *var* and *rifin* genes can be problematic, given the high sequence complexity in these genes and the potential cross-reactivity among microarray probes that target them. Thus, we hesitate to draw conclusions that implicate specific *var* or *rifin* genes as correlates of AR. We also emphasize that most AS biclusters do not contain members of these gene families and are associated with other cellular processes. The strong association observed between artemisinin sensitivity and genes responsible for antigenic variation and erythrocyte–host interactions may be a result of those processes being some of the most well-studied in *P. falciparum*, given their clinical relevance. In contrast, many *P. falciparum* proteins remain functionally uncharacterized and there may be critical, undiscovered gene regulatory pathways that cannot yet be revealed by functional enrichment analysis and thus remain occult in our assessment of the functional landscape of AS biclusters. AS biclusters that show more fragmented functional profiles may in fact represent key gene regulatory programs in the parasite that have yet to be characterized and aggregated into broader functional categories. The biclusters we generated for this study, because they implicate proteins in related functions, can offer a potential guide for functionally characterizing the parasite's less-studied proteins. Future research focused on characterizing these proteins and the pathways in which they participate will be critical for a global understanding of how *Plasmodium* parasites regulate phenotypic changes throughout their life cycle and in response to drug pressure.

## DATA AVAILABILITY

Transcriptomic data sets used to train and validate the *Pf*EGRIN model were published previously and are publicly available in the Gene Expression Omnibus (GEO) database. The training data set is available under GEO accession number GSE59097. Validation data sets are available under accession numbers GSE59098, GSE83667, and GSE116341.

## Supplementary Material

gkaa1245_Supplemental_Files

## Notes

Present address: Samuel A. Danziger, Bristol Myers Squibb, Seattle, WA, USA.
