# Supplementary material for: A systems-level gene regulatory network model for Plasmodium falciparum
Source: Nucleic Acids Res. 2021 Jan 15;49(9):4891–906. doi: 10.1093/nar/gkaa1245 (PMC8136813; doi:10.1093/nar/gkaa1245)
Supplement: gkaa1245_Supplemental_Files [file gkaa1245_supplemental_files.zip › SupplementaryFigures.pdf]

Supplementary Figures

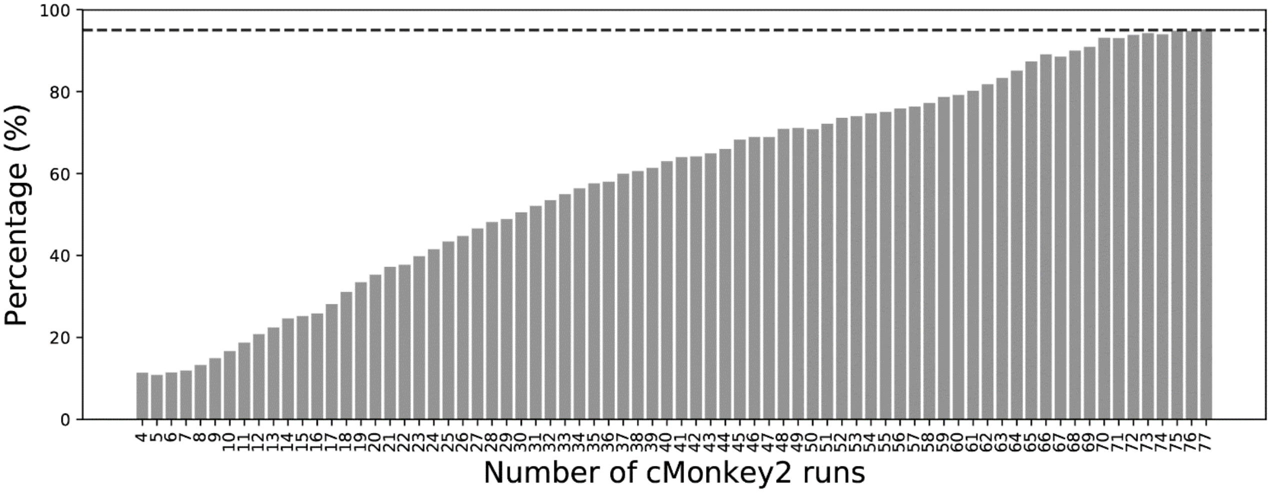

Figure S1. Convergence of average TR-target weights over 77 cMonkey2/Inferelator runs. Bars indicate the percentage of TR-target pairs with weights that differed less than 5% between *n* and *n*-3 cMonkey2 runs, with *n* given on the x-axis. Dashed line indicates 95%.

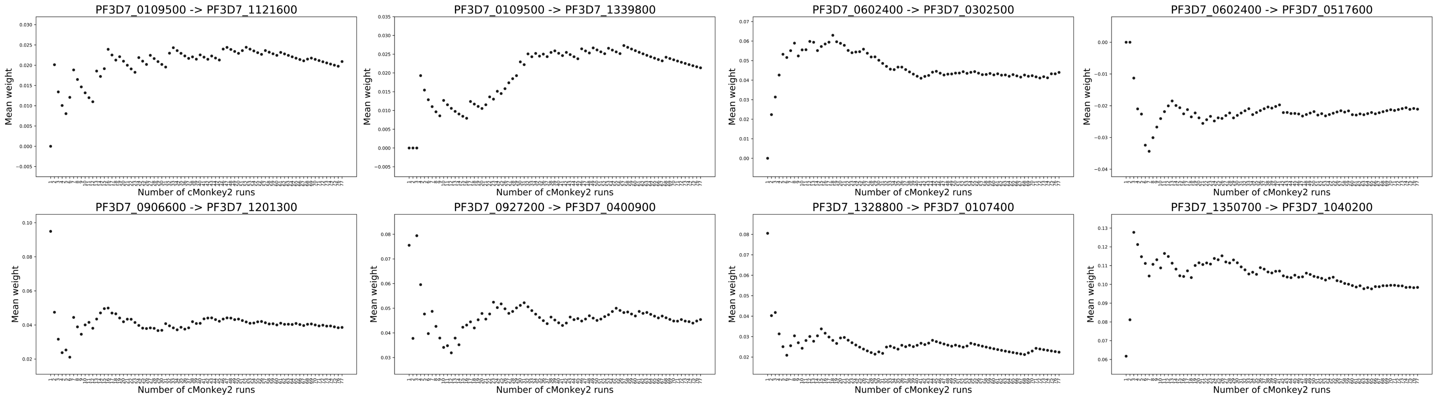

Figure S2. Examples of specific mean TR-target weights over 77 cMonkey2/Inferelator runs.

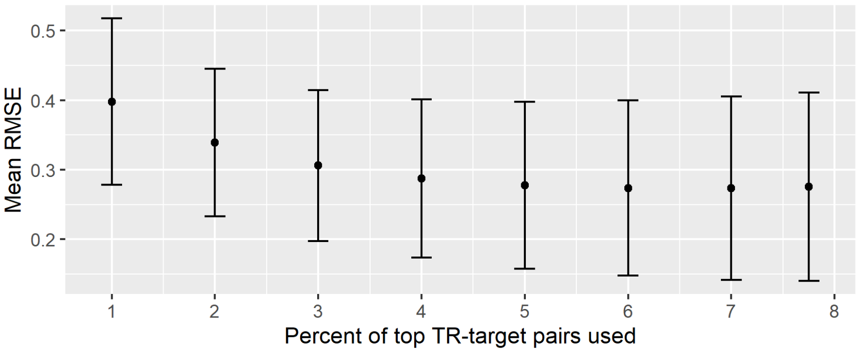

Figure S3. Mean RMSE values for model fits to training data versus percent of top-weighted TR-target pairs used in the *Pf*EGRIN model. Model fits were assessed using gene sets from the 38,500 biclusters generated to build the model. An optimal RMSE value was found using the top 7% of pairs.

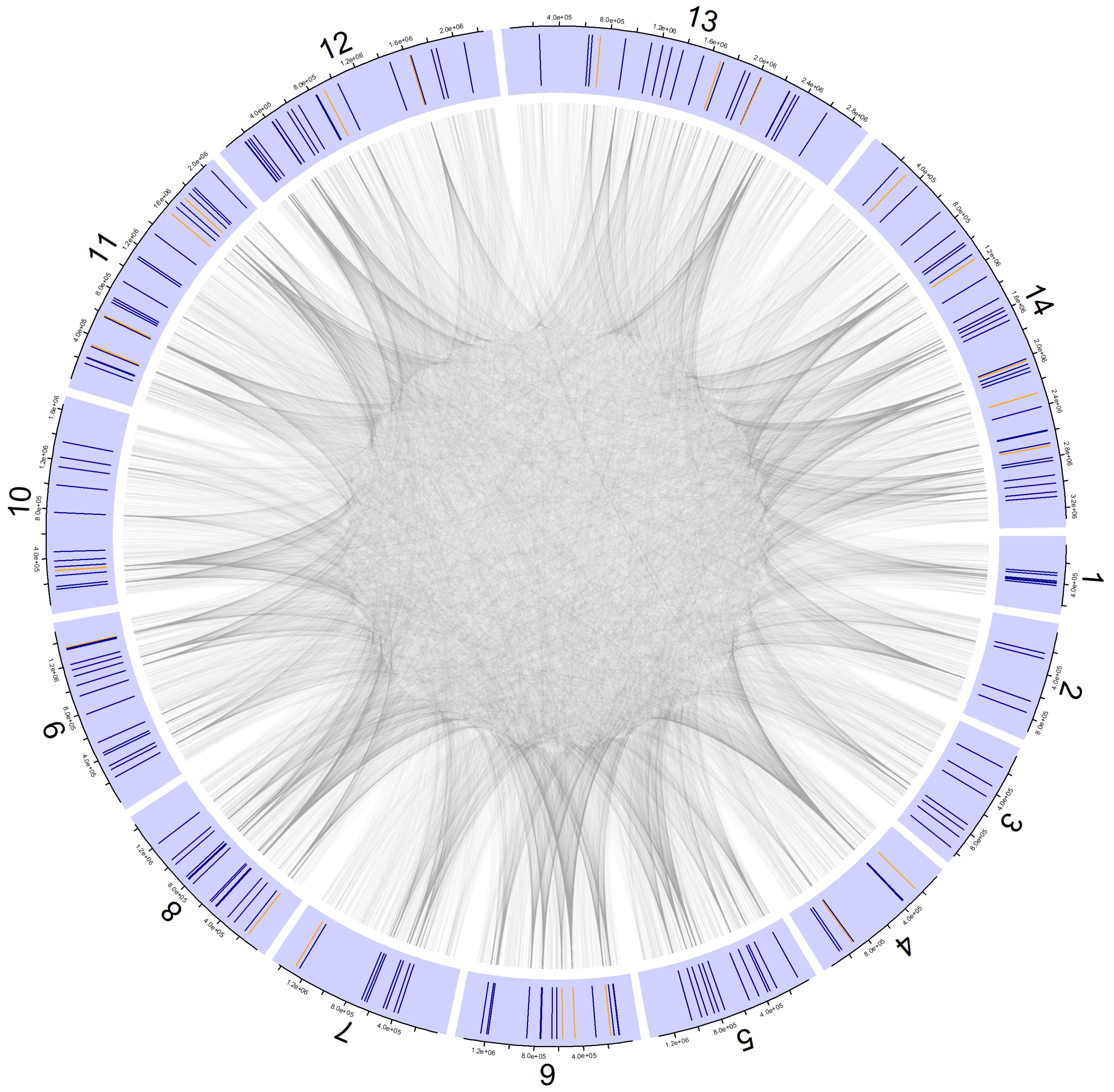

Figure S4. Visualization of gene regulatory interactions across the *P. falciparum* genome. Segments of the ring represent *P. falciparum* chromosomes. Lines inside segments indicate genomic location of TRs used in the *Pf*EGRIN model. Orange lines indicate ApiAP2 genes; blue lines indicate other TRs. Edges indicate TR-target interactions positioned according to the genomic location of interacting genes. For visualization purposes, only edges with absolute weights within the top quartile are shown.

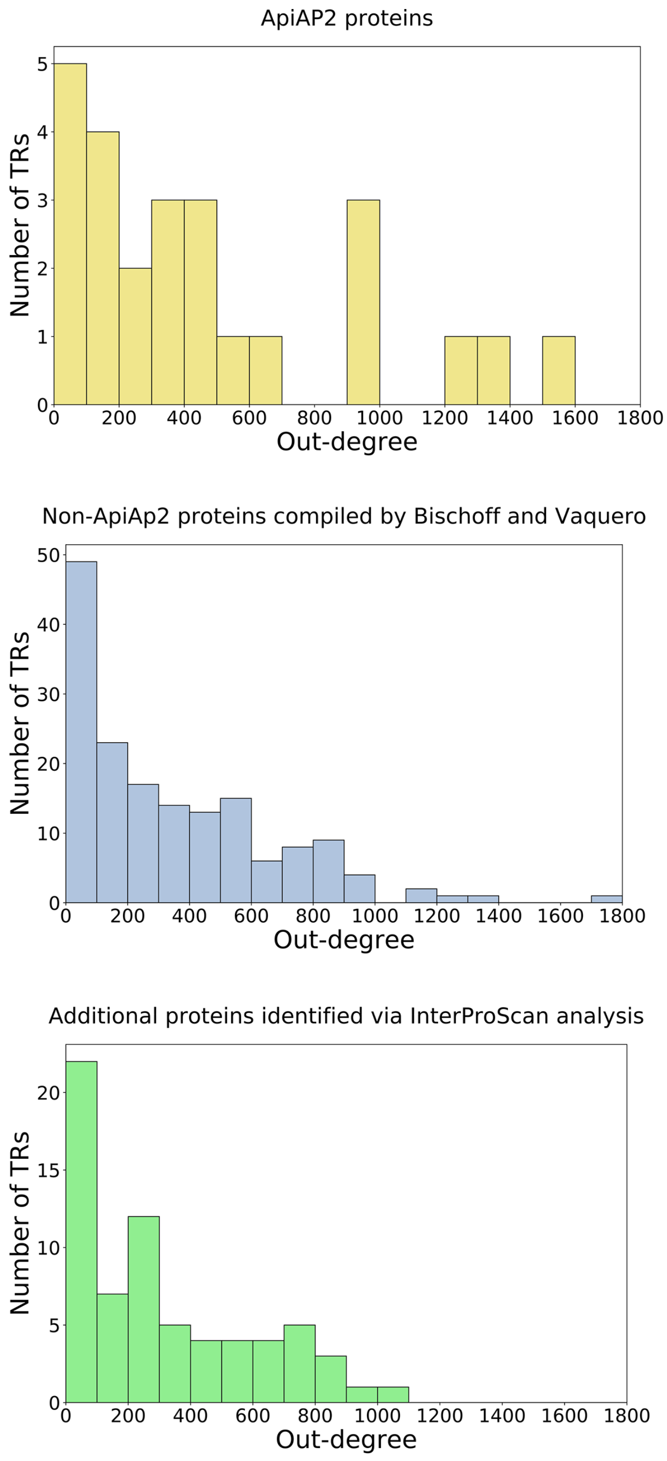

Figure S5. Distribution of out-degrees (number of predicted regulatory targets) among different subsets of TRs in the *Pf*EGRIN. *Top*: Out-degree distribution among Apicomplexan Apetala (ApiAP2) proteins. *Middle*: Out-degree distribution among regulatory proteins compiled by Bischoff and Vaquero (1) that are not ApiAP2 proteins. *Bottom*: Out-degree distribution among additional regulatory proteins included in the model based on results from our InterProScan analysis.

References

1. Bischoff,E. and Vaquero,C. (2010) In silico and biological survey of transcription-associated proteins implicated in the transcriptional machinery during the erythrocytic development of Plasmodium falciparum. *BMC Genomics*, **11**, 34.
